# Development and validation of the birth integrity questionnaire for measuring attitudes, maternity care, and perceptions of birth

**DOI:** 10.1186/s12884-025-08331-3

**Published:** 2025-10-27

**Authors:** Stephanie Batram-Zantvoort, Céline Miani, Oliver Razum, Manuel Batram

**Affiliations:** 1 Department of Epidemiology and International Public Health, School of Public Health, Bielefeld University, Bielefeld, Germany; 2 Sexual and Reproductive Health and Rights Research Unit, Institut National d’Études Démographiques (Ined), Aubervilliers, France; 3 Vandage GmbH, Bielefeld, Germany

**Keywords:** Birth integrity, Instrument validation, Quality of care, Obstetric violence, Maternity care, Birth experiences, Respectful maternity care

## Abstract

**Background:**

Public health research on maternal health has increasingly focused on care quality, emphasizing subjective evaluations and addressing issues such as obstetric violence and mistreatment during childbirth. In 2014, the WHO called for research to prevent and eliminate mistreatment, advocating for respectful maternity care. While significant research exists in Africa, Asia, and Latin America, research in European settings is lacking. Our Germany-based study on Birth Integrity aims to capture a holistic understanding of the birthing process, encompassing culturally mediated attitudes, actual birth experiences, and perceptions of childbirth.

**Methods:**

The Birth Integrity Questionnaire (BI-Q) was developed through construct definition, item development, expert reviews, and cognitive interviews. Psychometric properties were tested using exploratory factor analysis and structural equation modeling with 1,729 participants recruited via an anonymous, openly available, voluntary, non-incentivised online survey. Data analysis included reliability testing and examination of correlations between latent constructs.

**Results:**

The BI-Q demonstrated high reliability and validity, with 65 items across eleven latent constructs. The latent outcome construct of Birth Integrity, consisting of 22 items, was also successfully validated. Key influences on birth integrity included CONSENT, RESPECT, SUPPORT, and CARE. High standards of maternity care in Germany likely explained the lack of significant direct effect from constructs such as FACILITY, PRIVACY, INFORMATION, and RIGHTS, though these had indirect effects. GENDER, MEDICALIZATION, and BIRTH WISHES showed indirect effects as well, whereas DISCRIMINATION was omitted in our model due to statistical reasons.

**Conclusions:**

The BI-Q is a validated and reliable tool for measuring birth integrity. Findings highlight the significant impact of healthcare providers’ actions and attitudes on birth perception, advocating for improved maternity care and prioritizing women’s autonomy. The omission of DISCRIMINATION underscores the need to re-test the BI-Q in more diverse samples.

**Supplementary Information:**

The online version contains supplementary material available at 10.1186/s12884-025-08331-3.

## Introduction

### Background

Public Health research on maternal and newborn health has changed in the last decade. The traditional focus on birth outcomes, like mortality, morbidity, or cesarean section rates, has broadened to include a more comprehensive set of indicators. This shift considers the healthcare system’s conditions and processes that impact maternal and newborn outcomes [1]. In parallel, maternal health metrics have evolved to prioritize the quality of maternity care provision to gain deeper insights into the factors that shape a woman’s birth experience [2]. This expanded perspective has given rise to a distinct research area that centers on a patient-centered evaluation of maternity care provision. Initial studies exploring obstetric violence [3], disrespect and abuse [4], or mistreatment [5] during facility-based childbirth have gained substantial recognition and gave rise to investigations into this facet of gender-based and reproductive violence against women^1^. In 2014, the World Health Organization (WHO) called for intensified research and action to prevent and eliminate mistreatment of women during childbirth [6]. Since then, WHO has advocated for enhanced maternity rights [7, 8] and supported the development of the Respectful Maternity Care Charter (RMC) [9].

Extensive research on respectful care has been conducted in African and Asian countries (e.g [10–16]).,, and in Latin and South America, obstetric violence is a subject of social and political debate, with some countries even incorporating it into legislation [17–21]. In contrast, the topic of disrespect and abuse during childbirth remains a nascent area of research in the Global North, where our own research is located. However, activist movements have successfully brought attention to improper practices in obstetric care, and the reports from those affected, often self-describing their “birth trauma,” have begun to receive recognition, bringing the conditions of maternity care to the forefront and attracting the interest of the European research community.

Recent quantitative studies conducted in several European countries, including Poland [22], Spain [23–25], France [26, 27], Italy [28–30], Switzerland [31], Germany [32] and a multi-country study spanning 12 Regions within the WHO European Region [30] stress the need to thoroughly investigate the factors influencing women’s childbirth experiences in well-equipped healthcare settings. The findings from these studies underscore that issues related to consent, interventionism, impersonal care, emotional pressure, or discrimination have a substantial impact on women’s birthing experiences. We aim to develop a measure that serves to capture a holistic understanding of the birthing process from the perspective of the individual giving birth, including culturally mediated attitude towards childbirth, the actual experiences with birth-related care, and the perception of the birthing process.

### Adapting a feminist research methodology

A key feminist principle guiding our research is the emphasis on valuing lived experiences [33] by adopting an embodied understanding of care and prioritizing the emotions and thoughts elicited during childbirth. Existing measures, such as maternal satisfaction or mere descriptions of birth, may fall short in capturing the nuanced nature of childbirth. To conceptually differentiate between culturally mediated attitudes towards births, birth experiences (i.e., what happens intersubjectively during birth and external conditions surrounding birth) and the individual perception of one’s birth, we introduce the concept of birth integrity.

### Birth integrity

We define birth integrity as the holistic, deeply personal perception of childbirth, encompassing emotional, psychological, and physical dimensions [34, 35]. Birth integrity acknowledges the importance of women’s autonomy, dignity, agency, and overall well-being during childbirth. While birth integrity as a concept is meant to reflect the holistic and individually subjective way of how a woman perceives her birth, we seek to understand the impact of (culturally mediated) attitudes towards childbirth norms (e.g., on medicalization and gender) and the experiences with maternity care on one’s birth integrity (i.e. perception of birth).

### Aims and objectives

We aim to create and validate a theoretically sound instrument which we call the Birth Integrity Questionnaire (BI-Q). This questionnaire will gauge how normative attitudes toward birth and birth experiences shape an individual’s perception of childbirth. Expanding upon prior research, the BI-Q aims to illustrate the relationship between birth attitudes, birth experiences, and their impact on one’s perception of childbirth. This paper reports on the design, validation, and psychometric testing of the BI-Q. More specifically, our objectives are as follows:

- *Report on the development of the BI-Q factors (preliminary work).*
- *Evaluate the psychometric properties of all factors.*
- *Statistically examine the impact of birth-related factors (birth experiences and birth attitudes) on birth integrity.*

## Methods

For the method section, we will report on preliminary steps first (construct definition, item development, instrument judgement and refinement) and then describe the data collection and statistical analysis used for testing the psychometric properties of the BI-Q. All studies conducted as part of the scale development were ethically approved by the Bielefeld University ethics committee (Nr. 2021 − 162, Nr. 2022 − 106).

Construct definition.

### Theoretical framework and critical review

We grounded our concept of birth integrity in a theoretical paper [35] that examined childbirth through the lenses of risk theory and medicalization [36–38], embodiment [39], and intersectionality [40] exploring how structural, environmental, social, and individual factors contribute to health inequalities and intersecting forms of discrimination based on race, gender, and class. In parallel, in a critical review, we systematically analyzed 82 studies related to obstetric violence and related concepts using a meta-ethnographic approach, including reciprocal translation, in-line argumentation, and higher-level synthesis to propose the birth integrity multilevel framework [34]. As part of our methodological preparation for scale development, we additionally first extracted all items the included studies used to assess birth-related issues, which we then organized into overarching themes. We finally created a six-field birth integrity framework, ranging from macro to micro levels. This framework provides a valuable analytical distinction, elucidating the interconnected factors that influence both the birthing situation and the integrity of those undergoing childbirth.

### Item and scale generation

Building upon prior work (theoretical framework and critical review), in this phase we established twelve domains of interest, resulting in a total of 174 items. Our scale aimed to assess two facets of birth integrity: the (potential) factors influencing birth integrity (dependent variables) and birth integrity itself (outcome measure). Birth integrity (domain 13) is theoretically grounded in embodiment theories [41]. Measures related to birth integrity are the labor agentry scale [42], the birth satisfaction scale [43], the labor and delivery satisfaction index [44], and support and control in childbirth [45]. At this stage, eleven domains were established to reflect potential factors influencing birth integrity (domains 1–2 and 4–12). Domains 1 and 2 refer to prevailing discourses and practices around childbirth medicalization [46–48], risk perception [37, 38, 49–54] and gendered attitudes [55], with specific adaptations for the context of childbirth, including gendered shame [56, 57]. Domains 4–12 were conceptually framed to capture core dimensions of person-centered maternity care and the relational, environmental, and procedural aspects of childbirth that influence birth integrity. These domains are anchored in well-established concepts such as birth satisfaction [58], respectful maternity care [59], person-centered care [14], obstetric violence [23], disrespect and abuse in childbirth [60], mistreatment during facility-based childbirth [61], or discrimination during childbirth hospitalization [62]. They include the availability and responsiveness of healthcare providers, the provision of respectful, consensual, and supportive care, privacy, information sharing, and the upholding of patients’ rights. By linking these domains to both positive (e.g., satisfaction, person-centered care) and negative (e.g., obstetric violence, mistreatment) dimensions of maternity care, they capture the mechanisms through which the healthcare environment and provider behaviors shape a birthing person’s perceived integrity. Drawing from a rich pool of sample items extracted during the critical review, we adapted them to our purpose [15, 63–65]). Domain 3 was established in a later phase of the item and scale development process.

### Instrument judgement and refinement

#### Expert study I

We conducted an expert study to gather feedback on the domains and items through an online survey using evasys software [66]. We invited experts via email, encouraging them to share the survey link within their professional networks. Eligible experts included health professionals in maternity care (e.g., midwives, obstetricians), specialists in maternal mental health (psychotherapists and psychologists), social workers offering support to new mothers and crisis intervention, lactation consultants, birthrights activists, and public health experts working on maternal health. A total of 21 experts provided qualitative feedback on the BI-Q instrument. Experts used “ok” to indicate agreement with an item or offered critiques and suggestions through an open comment field. Comments were reviewed and used to refine items by improving wording, specifying content, merging similar items, or omitting where necessary.

#### Instrument construction II: expert study II

We conducted a content validity study with a second expert panel to gather judgmental evidence. This study ensures that the measurement domains effectively capture the intended content by assessing how well an item reflects the content, providing insights into clarity and representativeness of items [67–70]. Eligible experts were selected based on the criteria established for expert study I. To enhance the participatory nature of our research and align it with the genuine needs of the study population for the BI-Q, we additionally considered women with an own history of childbirth as experts by experience (lay experts) [71]. In all, 87 participants assessed the items for relevancy, essentiality, and clarity on a 1–4 rating scale for relevancy and essentiality, and a yes-or-no scale to rate whether the item was clear. A free comment section allowed additional thoughts and suggestions. Participants were free to skip an item if they felt they had insufficient knowledge. Content validity index (CVI) VI, Kappa statistic, and Content validity ratio (CVR) were computed for content validity quantification [68, 70, 72–75]. CVI encompasses two forms, one in relation to the item (item-level content validity index = I-CVI) and one regarding the scale (scale-level content validity index based on the average method = S-CVI/Av). While item clarity (Quality) is not necessarily part of content validation, we included it to identify items needing linguistic improvement. The free comment section facilitated a feedback loop for enhancement, such as phrasing adjustments, item order, and explanatory examples for better understanding. Indices, definitions and formular used for content validity can be found in Supplementary File 1.

The I-CVI for all items in the domains 4–12 and 13 scored 0.78 or higher; S-CVI scored above 0.90, indicating high content validity, supported by Kappa statistics higher than 0.74 for all items. For CVR, all items in the delineated domains scored higher than 0.30, meaning that experts considered them to be essential. In domains 1 and 2 (medicalization and gender norms), a few items scored slightly lower than 0.78 for I-CVI, indicating the need for revising them. However, CVR indicated essentiality for each item except one, which was subsequently dismissed. Overall, content validity for the BI-Q has shown to be excellent, except for the two dimensions mentioned (1 and 2). These exceptions seem not surprising as rating attitudes related to societal norms (i.e., medicalization, gendered norms) require a certain degree of familiarity with how societal discourses affect individuals’ attitudes [76].

#### Instrument construction III: cognitive interviewing

Cognitive interviewing is an evidence-based method for testing and refining instruments (to improve face validity). It focuses on identifying and improving issues related to question clarity, wording, and structure to enhance instrument validity and reliability [77–79]. Two participants independently read the items aloud and shared their thought processes (‘thinking-aloud’) while doing so. Cognitive interviews were recorded using digital devices, supplemented with interviewer observational notes. These notes highlighted items that did not fulfill their intended purpose or captured noticeable physical reactions from interviewees, such as facial expressions. After the interviews, items with unclear wording or purpose were identified by reviewing the audio recordings and interviewer notes, and were subsequently refined.

#### Final questionnaire for psychometric testing

Participant feedback from expert studies I and II, as well as the cognitive study, underscored the need for additional items specifically addressing birth preferences. In response, we introduced a new dimension (Domain 3). Furthermore, to enhance clarity and address recommendations for improved differentiation, we divided items related to experiences with healthcare professionals into two distinct categories: one for midwifery care and another for care provided by medical doctors (domains 5–10).

### Psychometric evaluation

#### Sample description and data collection

Women and individuals aged older than 18 who had given birth between January 1 st 2012 and December 31th 2022 to a newborn after at least 24 weeks of pregnancy in a German healthcare facility and were accompanied by a healthcare professional were eligible to participate in this study (exclusion criteria: unaccompanied birth, e.g., in a car). The structured questionnaire was available online via evasys survey software [66], with participation being anonymous, voluntary, and without incentives. Those with multiple birth experiences were encouraged to complete the questionnaire multiple times. The survey was promoted on social media, with parent initiatives, activist groups, and women’s health networks sharing it via email and social platforms. Posters with study details and a QR code were displayed in relevant locations like birth and family centers. Available in German, the survey run for six months (July-December 2022). Participants were fully informed about the study’s aims, design, duration, and data protection regulations (DSGVO) before providing consent. No personal information was collected.

For psychometric testing, we excluded participants who had undergone planned cesarean sections, as these typically occur before labor and are more similar to planned surgeries. Our focus was on collecting insights into care during labor and birth, so we included only those with experiences of vaginal births, vaginal-instrumental births (e.g., forceps), and unplanned or emergency cesarean sections. Births outside hospitals or maternity wards (e.g., home births, birth centers) were also excluded from psychometric testing to minimize missing data for certain domains (e.g., FACILITY).

#### Data analysis

The data was recoded to ensure all items had consistent polarity (highest value for the best outcome), except for the consent scale, which had only two options (“yes/no”). The dataset was then checked for missing values, outliers, and implausible answer patterns.

Subsequently, the dataset was randomly split into two data sets of equal size to ensure that the pruning steps did not interfere with the statistical inference of the structural equation model (SEM). The first data set was used to fit the exploratory factor analysis (EFA) and prune items with weak and/or cross-loadings. Using the findings from the EFA step, structural equation modelling (SEM) was fitted on the second half of the data set. Based on this initial SEM, necessary modifications were made to the structural model. Final results were computed on the full data set using the final functional form of the SEM. Item-factor correlations from the measurement model of the SEM were used to confirm the factor structure; therefore, a separate CFA was not conducted. Data analysis was performed using AMOS 26, R 4.3.2, and SPSS Statistics 29.

#### Exploratory factor analysis

EFA was performed to identify whether the items of a domain can be explained by one underlying factor (‘latent construct’) and to further reduce the number of items. In our case, and as we had built domains from literature and theory, we aimed to test the structure of each construct hence, separate analysis was performed for the item parcels of each construct. Principal axis analysis was used and factors with Eigen values (EV) larger than 1 were extracted.

For sample sizes larger than *n* = 350, factor loadings higher than 0.30 are considered relevant. As almost all items showed factor loadings higher that 0.30 – and therefore did not lead to the intended reduction of items – we decided to use a higher cut-off value [80]. Therefore, only items with absolute factor loadings larger than 0.45 on the first unrotated factor and without cross-loadings were retained. Items were excluded due to cross-loading when the absolute difference in factor loadings between the highest-loading and subsequent factors was 0.2 or lower. This rather rigid selection process ensured that only highly relevant items are part of BI-Q. Missing data was handled by pairwise exclusion, which allows us to maximize the use of available data. The Bartlett’s test and the Kaiser-Meyer-Olkin index were used to assess the applicability of EFA [81].

#### Structural equation modelling

While conventional approaches to scale development primarily focus on individual relationships between a construct (validated in earlier steps) and existing measures through regression analysis, utilizing SEM for scale development enables the exploration of a multitude of relationships between constructs and observable variables [82, 83]. In essence, SEM integrates elements from exploratory and confirmatory factor analysis, regression analysis, and pathway analysis. A SEM with a latent variable for each domain was fitted in AMOS 26. The latent constructs were used to explain the latent (outcome) factors for birth integrity. Due to the missing values, estimation was performed using full information maximum likelihood. Identification was ensured by setting the variances for all latent factors to 1 except for birth integrity. Due to it being an endogenous factor, the variance for birth integrity remained unfixed and instead the regression parameter for the first birth integrity item was fixed to one. The model fit was assessed using root mean square error of approximation (RMSEA) [82]. Cronbach’s alpha and Composite reliability score were computed from the SEM for the final item parcels [80, 84].

## Results

### Psychometric testing of the BI-Q

In Table 1, an overview on the different stages of the scale and items development process, psychometric testing through EFA, and testing of reliability of constructs is displayed. For the psychometric testing through EFA (*n* = 877) we used the Kaiser-Meyer-Olkin index (KMO) to assess the adequacy of the data for factor analysis [81]. For the four constructs SUPPORT (0.92), RESPECT (0.91), and BIRTH INTEGRITY (0.98), the KMO met the criteria *marvelous* with values higher than 0.90. Another five constructs have *meritorious* values, that are MEDICALIZATION (0.85), FACILITY (0.82), CARE (0.85), INFORMATION (0.83) and PRIVACY (0.84), whereas RIGHTS (0.71), BIRTH WISHES (0.70), and GENDER NORMS (0.78) show *average* values. The KMO for the constructs CONSENT (0.69) and DISCRIMINATION (0.67) were slightly less favorable, but still acceptable (*mediocre*). The Bartlett’s test rejected the hypothesis of a random correlation matrix for all domains, with all p-values < 0.001. Table 1 also displays the number of items eliminated through EFA due to either low factor loadings on the first factor or cross-loadings. The largest number of extracted factors was observed for DISCRIMINATION, which also had the lowest KMO and lowest explained variance. This is mainly an effect of the small number of participants reporting DISCRIMINATION and, hence, a large number of missing values. This also led to numerical difficulties in full maximum likelihood estimation of the SEM models, which is why the construct DISCRIMINATION was removed from further analysis.

**Table 1:** Phases of the validation process of the BI-Q, including number of items

Domains and latent constructs			Item and scale development process				Psychometric testing through EFA (*n* = 877)				Reliability of constructs		
			Initial item generation	Items after expert study I (*n* = 21)	Items after expert study II (*n* = 21)	Items after cognitive interviewing (*n* = 2)	Items after EFA	Number of extracted factors (Eigenvalue > 1)	Kaiser-Meyer-Olkin Test**	Total variance explained by factor 1 (%)	Items in final model	Cronbach’s alpha for item parcel	Composite reliability (CR) ***
Explanatory constructs	1	MEDICALIZATION	23	9	9	9	7	2	0.85	43.1	7	0.85	0.85
	2	GENDER NORMS	9	7	8	8	4	2	0.79	31.7	4	0.57	0.60
	3	BIRTH WISHES	0	0	6	6	4	1	0.71	45.9	4	0.73	0.78
	4	FACILITY	11	10	11	11	8	3	0.82	33.4	8	0.80	0.81
	5	CARE AVAILABILITY	22	21	19	15	5	2	0.85	54.4	5	0.88	0.88
	6	INFORMATION	8	6	7	7	6	1	0.83	52.3	6	0.85	0.84
	7	CONSENT*	13	13	3	3	3	1	0.69	70.2	3	n.a.	n.a.
	8	PRIVACY	7	6	5	5	5	1	0.84	71.7	5	0.89	0.90
	9	SUPPORT	14	13	10	10	9	1	0.92	72.6	9	0.95	0.95
	10	RESPECT	18	17	18	15	10	3	0.91	44.5	10	0.91	0.91
	11	RIGHTS	8	9	5	5	4	2	0.71	45.8	4	0.64	0.69
	12	DISCRIMINATION	10	11	10	10	8	4	0.67	27.2	removed	removed	removed
Outcome construct	13	BIRTH INTEGRITY	45	23	24	24	22	2	0.98	64.8	22	0.97	0.97

* 22 additional items for consent in specific interventions (not considered in EFA)

**KMO value interpretation: 0.90 and higher: marvelous. 0–89-0.80: meritorious. 0.70–0.79: average. 0.60–0.69: mediocre. 0.50–0.60: terrible. below 0.50: unacceptable

***A composite reliability (CR) between 0.60. (84) and 0.70 is recommended (81)

Based on the pruned item parcels, an SEM was constructed and fitted to the unused half of the data. One advantage of an SEM is the ability to test whether the relationship between the items and their respective construct is statistically significant. The SEM model, fitted on the half of the observations, which were not used for the EFA, confirmed that for all remaining item parcels the correlation with their respective construct is positive and statistically significant (*p* < 0.05) and, hence, no further pruning of items was performed. The only additional modification in this step was the construction of a sum scale for the consent items to match the Likert Scale of the other items. The scale was constructed to count the number of occasions where the woman was asked for consent and, hence given that there are four consent items, ranges from 0 to 4.

The final SEM model was fitted to all 1729 observations. The RMSEA for this final model is 0.064 (CI-90: 0.063, 0.064), which is a good (RMSEA < 0.05) to mediocre (RMSEA < 0.08) model fit, but comfortably under the cutoff for poor fitting models placed at RMSEA >0.10 [82]. As expected, all correlations between the items and their respective constructs were again positive and statistically significant (See appendix: Output for final SEM-Model), confirming the internal consistency of the constructs.

**Fig. 1:**
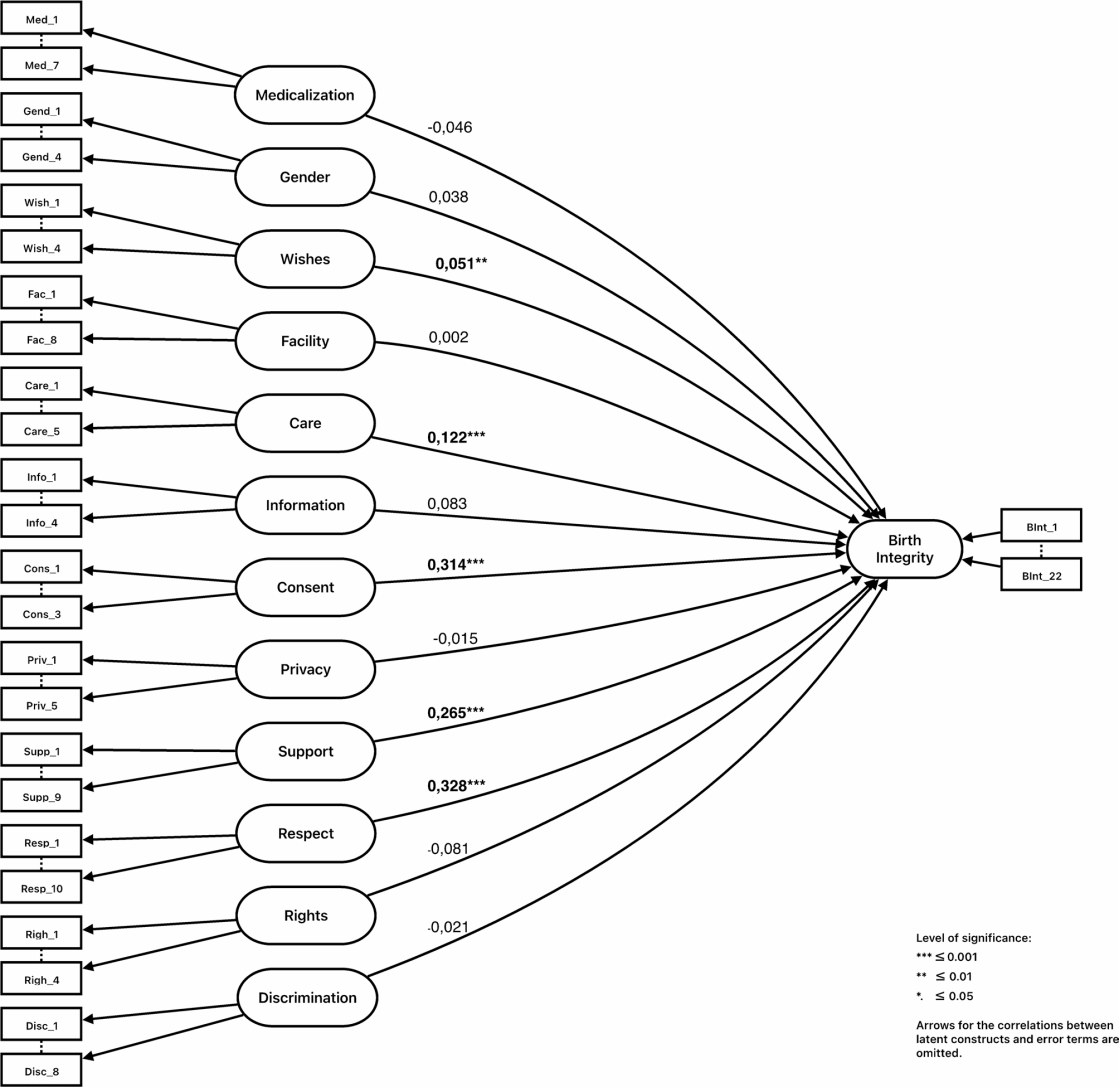
SEM-Model for the factors (constructs) influencing Birth Integrity

Figure 1 shows that the four constructs RESPECT, SUPPORT, CARE and CONSENT have a statistically significant effect on BIRTH INTEGRITY. The largest impact on BIRTH INTEGRITY with a standardized regression weight of 0.328 is observed for the construct RESPECT, followed by CONSENT (0.314), SUPPORT (0.265) and CARE (0.122). The coefficient for BIRTH WISHES is also significantly different from 0. However, with a standardized coefficient of 0.051, the impact of BIRTH WISHES is minuscule when compared to the other constructs. The p-values for INFORMATION, FACILITY, PRIVACY, MEDICALIZATION and GENDER are far larger than the threshold of 0.05, therefore showing that these constructs are highly unlikely to have an impact on BIRTH INTEGRITY for populations which are equivalent to the sample under analysis.

A translation of the final items in the construct of BIRTH INTEGRITY from German to English is displayed in Table 2 for exemplary reasons (non-validated in English). All validated items in German language as well as an English translation of these items (non-validated) can be found in the Supplementary File 2.

**Table 2:** Birth integrity construct, translated to English

	Question	Answer option	Inverted
1	During birth, I felt valued, respected and/or appreciated.	1 does not apply at all 2 does rather not apply 3 does partly apply 4 does rather apply 5 does fully apply 0 cannot/would like not to answer	
2	During birth, I put up with things or gave up, even though I felt inner resistance.		x
3	During birth, I felt safe, protected and/or in good hands.		
4	During birth, I felt disconnected, at times absent, dissociated and/or viewed myself from ‘on the outside’. *Attention: this does not mean a desirable, hormone-induced “high” or " cloudiness but a stress reaction that can occur when you feel a strong loss of control.		x
5	I felt belittled, degraded and/or dehumanized.		x
6	During birth, I experienced my boundaries being violated by others.		x
7	During birth, I was completely with myself, internally calm, and able to engage in the birthing process.		
8	During birth, I have developed severe nervousness, anxiety, despair and/or panic.		x
9	During birth, I felt lonely, alone, neglected, overlooked and/or considered unimportant.		x
10	During birth, I felt seen, heard and/or taken seriously.		
11	During birth, I felt shame.		x
12	During birth, I had enough control over the situation to have confidence in the actions of others. *Please note: this does not refer to control over the physical process of birth.		
13	During birth, I felt intimidated, speechless and/or at the mercy of others.		x
14	During birth, I felt harmed, attacked and/or as if I was left with no choice.		x
15	During birth, I felt burdened, distressed and/or sad.		x
16	During birth, I felt comfortable, happy and/or exhilarated’ by the birth hormones.		
17	Looking back at birth, I reproach myself.		x
18	Looking back at birth, I feel gratitude. well-being and/or satisfaction.		
19	Looking back at birth, I feel gratitude, a sense of well-being and/or satisfaction.		x
20	Looking back at birth, I feel stress, nervousness, anxiety and/or irritability.		x
21	Looking back at birth, I feel pride, amazement, and/or awe.		
22	Looking back at birth, I feel anger, rage and/or bewilderment.		x

*Definition of the direction: the higher the value*,* the higher/more preserved is birth integrity*

English version has been translated for exemplary reasons. The BI-Q has only been validated in German language

### Correlations of latent explanatory constructs

The SEM-model allowed for investigating correlations between the latent explanatory constructs (Table 3). Almost all relationships between the constructs were statistically significant except for BIRTH WISHES and GENDER NORMS, where almost no statistically significant correlations with other constructs were observed. Strong correlations are observed between almost all other constructs, ranging from 0.912 (between SUPPORT and INFO) to 0.488 (between PRIVACY and CONSENT), which is still considered a strong association. A model where the correlations were set to 0 had comparable results regarding the regression coefficients and their significance, which shows that the correlations and indirect effects do not shift the interpretation of the results.

**Table 3:** Correlation of latent explanatory constructs

	MEDICALIZATION/ RISK	GENDER NORMS	BIRTH WISHES	FACILITY	CARE AVAILABILITY	INFORMATION	CONSENT	PRIVACY	SUPPORT	RESPECT	RIGHTS
MEDICALIZATION/RISK		0.478	-0.572	0.096	0.083	0.183	0.284	0.156	0.166	0.168	0.098
GENDER NORMS	0.478		-0.189	0.046 (n.s.)	0.057 (n.s.)	0.096	0.167	0.046 (n.s.)	0.103	0.105	0.006 (n.s.)
BIRTH WISHES	-0.572	-0.189		0.034 (n.s.)	0.072	0.026 (n.s.)	-0.047 (n.s.)	-0.02	0.05 (n.s.)	0.025 (n.s.)	0.112
FACILITY	0.096	0.046 (n.s.)	0.034 (n.s.)		0.8	0.734	0.493	0.597	0.672	0.570	0.629
CARE AVAILABILITY	0.083	0.057 (n.s.)	0.072	0.8		0.826	0.544	0.551	0.788	0.646	0.649
INFORMATION	0.183	0.096	0.026 (n.s.)	0.734	0.826		0.691	0.644	0.912	0.794	0.766
CONSENT	0.284	0.167	-0.047 (n.s.)	0.493	0.544	0.691		0.488	0.804	0.694	0.854
PRIVACY	0.156	0.046 (n.s.)	0.05 (n.s.)	0.597	0.551	0.644	0.488		0.605	0.645	0.558
SUPPORT	0.166	0.103	0.05 (n.s.)	0.672	0.788	0.912	0.804	0.605		0.785	0.845
RESPECT	0.168	0.105	0.025 (n.s.)	0.570	0.646	0.794	0.694	0.645	0.785		0.681
RIGHTS	0.098	0.006 (n.s.)	0.112	0.629	0.649	0.766	0.854	0.558	0.845	0.681	

*n.s.* not significant (confidence level: 0.05)

### Initial results on birth integrity by participants characteristics and birth-related information

A total of 1,729 participants with childbirth experience were included to assess the psychometric properties of the BI-Q. Birth Integrity scores, ranging from 1 to 5, we calculated by participants’ characteristics and birth-related information. As shown in Tables 4 and 5, most participants were 25–35 years old (77.7%), held a university degree (62.9%), had German or EU citizenship (99.1%), were native speakers (96.6%), and identified with German ethnicity or cultural affiliation (89.3%). Nearly a third experienced psychological distress during pregnancy or birth (27.8%). The majority reported their first birth experience (79.6%), with over two-thirds giving birth between March 2020 and December 2022, during the COVID-19 pandemic. Higher birth integrity scores were associated with older age (mean: 3.19, SD: 1.07 for participants under 25 vs. mean: 3.82, SD: 0.795 for those 36 and older), higher education, multiparity, and the absence of psychological distress (mean: 3.87, SD: 0.820 vs. 3.49, SD: 0.866).

As shown in Tables 4 and 68.2% of participants had vaginal births, 12.6% had instrumental vaginal births, 16.5% underwent unplanned cesareans after labor began, and 2.6% had emergency cesareans. Among those with vaginal births, 72.2% experienced neither fundal pressure nor episiotomy, while only 25.7% of those with instrumental births reported no such interventions. Vaginal births had the highest birth integrity scores (3.96, SD: 0.766), with all other birth modes showing lower values (3.34 SD: 0.909–3.30 SD: 0.861). Lower birth integrity was observed when episiotomy or fundal pressure was used. An obstetrician was present for 86.2% of vaginal and instrumental births, with these participants showing lower birth integrity (3.84, SD: 0.831) compared to those attended only by a midwife (4.10, SD: 0.650). A companion of choice was present for 82.0% of participants, and 3.9% of those who gave birth during COVID-19 were required to wear a mask throughout, with 23.8% masking for part of the time. Having a companion was associated with higher birth integrity (3.82, SD: 0.831) compared to those without (3.41, SD: 0.910). Mask-wearing during labor was linked to lower birth integrity (3.32, SD: 0.978) compared to those who did not wear a mask (3.97, SD: 0.718).

**Table 4:** Values for birth integrity by participants characteristics, Germany, 2022 (*n* = 1729)

	*N*	%	Birth integrity* mean (SD)
Overall			
	1729	100.0%	3.76 (0.850)
Age			
18–24	90	5.2%	3.19 (1.07)
25–35	1344	77.7%	3.79 (0.832)
36–42	293	17.0%	3.82 (0.795)
43 or older	2	0.1%	4.31 (0.204)
Parity			
Primipara	1377	79.6%	3.70 (0.857)
Multipara	352	20.4%	4.02 (0.773)
Year of childbirth			
2012- 02/2020	507	29.3%	3.52 (0.944)
03/2020–10/2020	182	10.5%	3.64 (0.896)
2021	406	23.5%	3.82 (0.797)
2022	634	36.7%	3.96 (0.728)
Psychological distress during pregnancy/around time of birth			
yes	481	27.8%	3.49 (0.866)
no	1248	72.2%	3.87 (0.820)
Education			
Secondary school (low)	8	0.5%	3.42 (0.972)
Secondary school (middle)	157	9.1%	3.58 (0.975)
Highschool	366	21.1%	3.67 (0.908)
University degree	1088	62.9%	3. 81 (0.808)
Doctoral degree	110	6.4%	3.97 (0.764)
German or EU-citizenship			
yes	1714	99.1%	3.77 (0.849)
no	15	0.9%	3.19 (0.845)
German language native			
yes	1670	96.6%	3.77 (0.845)
no	59	3.4%	3.47 (0.934)
German ethnicity and/or cultural affiliation*			
yes	1544	89.3%	3.78 (0.847)
yes, among other	64	3.7%	3.78 (0.773)
no	121	7.0%	3.54 (0.905)

* Participants were asked how others usually would describe their ethnicity/cultural affiliation

**Table 5:** Values for birth integrity, by birth-related information, Germany, 2022 (*n* = 1729)

	*N*	*n*/*N*	%	nn/*n*	%	Birth integrity* mean (SD)
Birth modes and interventions	1729					
Vaginal		1180	68.2%			3.96 (0.766)
none				852	72.2%	4.06 (0.694)
Episiotomy				116	9.8%	3.88 (0.771)
Fundal pressure				108	9.2%	3.63 (0.921)
Episiotomy AND fundal pressure				85	7.2%	3.52 (0.878)
Episiotomy OR fundal pressure**				19	1.6%	3.50 (0.958)
Instrumental vaginal birth		218	12.6%			3.34 (0.909)
none				56	25.7%	3.94 (0.672)
Episiotomy				34	15.6%	3.47 (0.821)
Fundal pressure				51	23.4%	3.31 (0.917)
Episiotomy AND fundal pressure				72	33.0%	3.08 (0.947)
Episiotomy OR Fundal pressure**				5	2.3%	3.68 (0.722)
Unplanned c-section***		286	16.5%			3.30 (0.861)
Emergency c-section****		45	2.6%			3.30 (0.775)
Obstetrician present at vaginal and vaginal-instrumental birth	1398					
yes		1205	86.2%			3.84 (0.831)
no		193	13.8%			4.10 (0.650)
Companion of choice	1722					
not present		164	9.5%			3.41 (0.910)
partly present		146	8.5%			3.65 (0.869)
present		1412	82.0%			3.82 (0.831)
Mask mandate	1218					
mostly		48	3.9%			3.32 (0.978)
partly		290	23.8%			3.67 (0.865)
never		880	72.2%			3.97 (0.718)

*higher values present more positive birth integrity (values between 1–5)

**AND missing value on episiotomy OR fundal pressure

***unplannend c-section: after the onset of labor

****emergency c-section: after the onset of labor with high urgency

## Discussion

Our study introduces a validated scale specifically measuring birth integrity and is the first to link birth-related experiences with subjective birth perceptions. Content validity was established through expert evaluations and cognitive interviews. Our primary objective was to validate the latent constructs within the Birth Integrity Questionnaire (BI-Q), with Exploratory Factor Analysis (EFA) and Structural Equation Modeling (SEM) supporting its validity. After item reduction, 65 items across eleven latent constructs were confirmed in the German version of the BI-Q, including a validated 22-item construct for Birth Integrity.

Our second objective was to assess the impact of various birth-related factors on birth integrity. SEM identified four factors—CONSENT, RESPECT, SUPPORT, and CARE—as statistically significant influences. These findings align with prior research, highlighting the substantial effect of maternity care providers’ actions and attitudes on parturient experiences [24, 85–87]. The BI-Q validation establishes birth integrity as a robust measure of the relationship between subjective birth perceptions and childbirth experiences.

The lack of a significant impact of FACILITY may be due to the high standard of maternity care in Germany, with well-equipped labor rooms, strict hygiene, and access to medication. Similarly, PRIVACY may not have shown an effect because German parturients typically experience high levels of privacy, such as private labor rooms, ensuring privacy and confidentiality. This contrasts with findings from a study on Jordanian women, where room-sharing and lack of privacy during birth led to feelings of dehumanization, embarrassment, and fear [88]. We conclude that, in our study’s context, high facility and privacy standards do not significantly impact birth integrity. However, these factors may play a more crucial role in other settings, indicating the need for context-sensitive measures of birth integrity.

While INFORMATION does not directly impact birth integrity, it strongly correlates with key constructs like CONSENT, SUPPORT, RESPECT, and CARE, suggesting it indirectly influences birth integrity through aligned actions such as informed consent. However, INFORMATION alone is insufficient to ensure high birth integrity. The same applies to RIGHTS.

Attitudes towards birth-related MEDICALIZATION and GENDER NORMS do not directly impact birth integrity. Although a medicalized, risk-oriented view of childbirth is the prevailing norm [89], the actual experience of obstetric interventions (as a potential proxy for medicalization) seem to reduce birth integrity. An Israeli study on attitudes toward medicalization and their association with planned and actual modes of birth (n = 836) found that favorable attitudes toward medicalization were associated with a higher likelihood of choosing or experiencing more medicalized modes of birth, both planned and unplanned [48]. This suggests that attitudes and experiences may have converged in our study population, diminishing their distinct influence on birth integrity. Further research is necessary to better understand how gender norms and power (im)balances influence labor room interactions [90] and labor ‘performance’ [91], which is crucial for comprehending the impact of gender norms on birth integrity.

Regarding BIRTH WISHES, there is a small yet statistically significant impact on birth integrity, possibly indicating that having specific wishes for birth influences its perception. This is further supported by the fact that the construct BIRTH WISHES (where a higher value indicates a greater desire for an intervention-free birth) negatively correlates with the constructs of MEDICALIZATION (where a higher value indicates a more medicalized and risk-related view on birth) and GENDER NORMS (where a higher value indicates approval of traditional gender-related norms in childbirth). Existing findings on birth-related attitudes, risk perception, and views on the medicalization of childbirth suggest a similar effect on childbirth experiences [48, 92, 93].

Further research should be conducted to understand the extent to which the latent constructs of MEDICALIZATION, GENDER NORMS, and BIRTH WISHES affect birth integrity when assessed before childbirth. A panel study design, rather than a cross-sectional study design, would be more effective for this purpose, which might explain the limited influence of birth-related attitudes and wishes on birth integrity observed in our study.

The omission of DISCRIMINATION from the final SEM model likely resulted from our homogeneous sample, which mostly consisted of highly educated, language-proficient participants with German or EU citizenship, culturally aligned with the study context. This likely excluded those who might experience discrimination. We stress that discrimination, racism, and stereotyping during childbirth remain significant issues, with affected individuals often facing severe consequences such as abandonment, verbal abuse, rough treatment, and sexism [28, 94–96].

## Concluding remarks

The Birth Integrity Questionnaire (BI-Q) measures a birthing person’s perceived and felt sense of integrity, including dignity, autonomy, and respect during childbirth. It is, to our knowledge, the first tool that can demonstrate how specific care practices, such as consented care or support, directly influence overall birth integrity. This integrative perspective provides both conceptual and practical value for research and clinical practice, enabling a holistic assessment of childbirth experiences. Future research should test the BI-Q in more diverse study samples (e.g., level of education, migrant backgrounds) with particular emphasis on examining the impact of discrimination during childbirth on birth integrity.

## Supplementary Information

Supplementary Material 1.

Supplementary Material 2.

## Data Availability

The datasets analyzed during the current study are not publicly available due data protection measures but are available from the corresponding author on reasonable request.

## Footnotes

1 We acknowledge that individuals with diverse gender identities, including nonbinary and transgender individuals, give birth. For this article, we use the terms woman/women, as the majority of individuals giving birth identifies as female.
