# Supplementary material for: Development and validation of the birth integrity questionnaire for measuring attitudes, maternity care, and perceptions of birth
Source: BMC Pregnancy Childbirth. 2025 Oct 27;25:1141. doi: 10.1186/s12884-025-08331-3 (PMC12560401; doi:10.1186/s12884-025-08331-3)
Supplement: Supplementary file 1 — Supplementary Material 1. [file 12884_2025_8331_MOESM1_ESM.docx]

**Supplementary file 1: Indices, definitions, and formulas for content validity of the BI-Q**

|  | |  |  |
| --- | --- | --- | --- |
| **Indices** | **Definition** | | **Formula** |
| N, n | Number of experts | | n.a. |
| A | Number of experts who agree that item is relevant | | n.a. |
| Ne | Number of experts who valuated the items essential | | n.a. |
| PC | The probability of chance agreement | | PC = [N! / A! (N-A)!] x 0,5N |
| Kappa | Consensus index of interrater agreement (degree of agreement beyond chance). Kappa values above 0.74 is considered to be excellent (0-74-0.6: good; 0.59- 0.4: fair) | | K= (I-CVI - PC) / (1 - PC) |
| CVR | Content validity ratio measures the essentiality of an item. CVR varies between -1 and 1, and a higher score indicates higher agreement between participants. Items scoring a CVR greater than 0.30 remain at the instrument (Lawshe | | (Ne-N/2)/(N/2) |
| S-CVI/Ave | The average of I-CVI scores across all items. An S-CVI/Ave of 0.90 or higher is considered excellent. | | S-CVI/Ave = (sum of I-CVI scores)/(number of items) |
| I-CVI | Item-level content validity index. I-CVI of 0.78 or higher is considered excellent. Items scoring between 0.70-0.78 need revision, and a value below 0.70 means that the item is eliminated. | | I-CVI = (agreed item)/ (number of experts) |
| Quality of item | Percentage of experts who considered item to be clearly worded and comprehensible | | n.a. (qualitative comments) |
