# Supplementary material for: Development and validation of the birth integrity questionnaire for measuring attitudes, maternity care, and perceptions of birth
Source: BMC Pregnancy Childbirth. 2025 Oct 27;25:1141. doi: 10.1186/s12884-025-08331-3 (PMC12560401; doi:10.1186/s12884-025-08331-3)
Supplement: Supplementary file 2 — Supplementary Material 2. [file 12884_2025_8331_MOESM2_ESM.docx]

| **Supplementary file 2: List of validated variables and constructs of the BI-Q, German language (and English translation, not validated)** | | | | | | |  |  |
| --- | --- | --- | --- | --- | --- | --- | --- | --- |
| **Construct: MEDICALIZATION** | | | | | | | *English translation (not validated)* | |
| **Variable** | | | **Inverted** | **Factor** | | |  |  |
|  |  |  |  | 1 | 2 | 3 |  |  |
| Med_1 | Der natürliche (physiologische) Geburtsprozess ist risikoreich. Daher ist die Anwesenheit von ärztlichem Fachpersonal* bei der Geburt wichtig.  * ärztliches Fachpersonal: Gynäkolog*in, Anästhesist*in | 1 stimme gar nicht zu 2 stimme eher nicht zu 3 stimme teilweise zu 4 stimme eher zu 5 stimme stark zu |  | 0,768 | -0,153 |  | The natural (physiological) birth process is risky. Therefore, the presence of doctors* during labour and birth is important.  * doctors: gynecologist, obstetrician, anesthesiologist | 1 strongly disagree  2 rather disagree  3 partly agree  4 tend to agree  5 strongly agree |
| Med_2 | Der natürliche (physiologische) Geburtsprozess ist risikoarm. Eine kontinuierliche Hebammenbetreuung reicht normalerweise aus. Sollte dennoch ärztliche Hilfe benötigt werden, wird die Hebamme dies rechtzeitig erkennen. |  | x | 0,665 | -0,368 |  | The natural (physiological) birth process is low-risk. Continuous midwife care is usually sufficient. However, if doctors are required, the midwife will recognize this in good time. |  |
| Med_3 | Geburten sollten möglichst interventionsfrei verlaufen (wenige Unterbrechungen und Eingriffe in den Geburtsverlauf, zum Beispiel: kein CTG-Schreiben*, keine PDA).  *CTG: Wehen- und Herztonschreiber |  | x | 0,746 | 0,032 |  | Births should be as intervention-free as possible (few interruptions and interventions in the birth process, for example: no CTG recording*, no epidural).  *CTG: labor and heart sound recorder |  |
| Med_4 | Geburten sollten mit den technischen Mitteln des Geburtsmonitoring 'überwacht' werden (z.B. dauerhaftes CTG-Schreiben). |  |  | 0,756 | 0,093 |  | Births should be 'monitored' using the technical means of birth monitoring (e.g. continuous CTG recording). |  |
| Med_5 | Wenn der errechnete Geburtstermin überschritten ist oder beim Kind ein sehr hohes oder sehr niedriges Gewicht vermutet wird, sollte die Geburt zeitnah eingeleitet oder ein Kaiserschnitt durchführt werden. |  |  | 0,551 | 0,120 |  | If the expected date of birth has been exceeded or the baby is suspected of having a very high or very low weight, the birth should be induced promptly or a caesarean section should be performed. |  |
| Med_6 | Außerklinische Geburtssettings (Geburtshaus, Hebammenpraxis oder Hausgeburten) sind sicher, sofern die Schwangerschaft problemlos und risikoarm verlaufen ist und keine medizinischen Gründe gegen eine außerklinische Geburt sprechen. |  | x | 0,669 | -0,118 |  | Out-of-hospital birth settings (birth center, midwife practice or home births) are safe as long as the pregnancy was problem-free and low-risk and there are no medical reasons against an out-of-hospital birth. |  |
| Med_7 | Klinische Geburtssettings (Krankenhäuser, Geburtsklinik) sind sicher, da dort die bestmögliche geburtshilfliche Versorgung für alle Gebärenden bereitgestellt wird. |  |  | 0,520 | 0,130 |  | Clinical birth settings (hospitals, maternity clinics) are safe because they provide the best possible obstetric care for all women giving birth. |  |
| *Definition der Richtung: Je höher der Wert, desto höher die Zustimmung zu medikalisierten Geburten/ Medikalisierung/ Risikowahrnehmung von Geburten* | | | | | | | *Definition of direction: The higher the value, the higher the approval of medicalized births/medicalization/risk perception of births* | |
|  |  |  |  |  |  |  |  |  |
| **Construct: GENDER NORMS** | | | | | | | *English translation (not validated)* | |
| **Variable** | | | **Inverted** | **Factor** | | |  |  |
|  |  |  |  | 1 | 2 |  |  |  |
| Gend_1 | Heutzutage machen Schwangere sich zu viele Gedanken um die Geburt. Gebärende vorheriger Generationen haben Geburten unter schlechteren Bedingungen und mit weniger "Wehwehchen" gemeistert. | 1 stimme gar nicht zu 2 stimme eher nicht zu 3 stimme teilweise zu 4 stimme eher zu 5 stimme stark zu |  | 0,553 | -0,198 |  | Nowadays, pregnant women worry too much about giving birth. Women giving birth in previous generations gave birth under worse conditions and with fewer “aches and pains”. | 1 strongly disagree  2 rather disagree  3 partly agree  4 tend to agree  5 strongly agree |
| Gend_2 | Die Gebärende sollte sich während der Geburt zurückhalten, um den Hebammen und dem ärztlichen Fachpersonal nicht zur Last zu fallen (z.B. Entscheidungen nicht in Frage stellen, nicht diskutieren). |  |  | 0,551 | 0,122 |  | The woman giving birth should keep a low profile during the birth so as not to be a burden on the midwives and doctors (e.g. do not question decisions, do not discuss). |  |
| Gend_3 | Die Gebärende sollte auf Anweisungen von Hebammen und ärztlichem Fachpersonal warten, statt selbst aktiv zu werden. |  |  | 0,496 | -0,077 |  | The woman giving birth should wait for instructions from midwives and doctors instead of taking action herself. |  |
| Gend_4 | Die Gebärende sollte nach der Geburt nicht mehr zu lang über den Schmerz und das, was während der Geburt passiert ist, nachdenken. Hauptsache ist, dass es dem Neugeborenen gut geht. |  |  | 0,539 | -0,218 |  | After the birth, the woman giving birth should not think too long about the pain and what happened during the birth. The main thing is that the newborn is doing well. |  |
| *Definition der Richtung: Je höher der Wert, desto höher die Zustimmung zu traditionellen Geschlechternormen* | | | | | | | *Definition of direction: The higher the value, the higher the approval of traditional birth-related gender norms* | |
|  |  |  |  |  |  |  |  |  |
| **Construct: BIRTH-RELATED WISHES** | | | | | | |  |  |
| **Variable** | | | **Inverted** | **Factor** | | | *English translation (not validated)* | |
|  |  |  |  | **1** |  |  |  |  |
| Wunsch_1 | Ich wünschte mir eine möglichst interventionsfreie Geburt. | 1 trifft gar nicht zu 2 trifft eher nicht zu 3 trifft teilweise zu 4 trifft eher zu 5 trifft voll zu  0 kann/ möchte ich nicht beantworten |  | 0,727 |  |  | I wanted the birth to be as intervention-free as possible. | 1 Does not apply at all  2 rather not applicable  3 partly true  4 Rather true  5 fully applies  0 I cannot/would not like to answer |
| Wunsch_2 | Ich wollte unbedingt eine PDA oder Spinalanästhesie (Betäubung von Rückenmarksnerven). |  | x | 0,631 |  |  | I really wanted an epidural or spinal anesthesia (anesthesia of the spinal nerves). |  |
| Wunsch_3 | Ich wünschte mir eine vaginale ('natürliche') Geburt. |  |  | 0,715 |  |  | I wished for a vaginal ('natural') birth. |  |
| Wunsch_4 | Ich wünschte mir einen Kaiserschnitt. |  | x | 0,755 |  |  | I wished for a caesarean section. |  |
| J*e höher der Wert, desto größer der Wunsch nach einer "natürlichen", "interventionsfreien" Geburt.* | | | | | | | *The higher the value, the greater the desire for a “natural”, “intervention-free” birth.* | |
|  |  |  |  |  |  |  |  |  |
| **Construct: FACILITY** | | | | | | |  |  |
| **Variable** | | | **Inverted** | **Factor** | | | *English translation (not validated)* | |
|  |  |  |  | **1** | **2** | **3** |  |  |
| Klinik_1 | Bei Ankunft in der Geburtsklinik wurde ich zeitnah aufgenommen und durch eine Hebamme betreut. | 1 trifft gar nicht zu 2 trifft eher nicht zu 3 trifft teilweise zu 4 trifft eher zu 5 trifft voll zu  0 kann/ möchte ich nicht beantworten |  | 0,734 | -0,350 | 0,376 | When I arrived at the maternity clinic, I was admitted promptly and looked after by a midwife. | 1 Does not apply at all  2 rather not applicable  3 partly true  4 Rather true  5 fully applies  0 I cannot/would not like to answer |
| Klinik_2 | Bei Ankunft in der Geburtsklinik glaubte man mir, dass meine Geburt im Gang ist/ ich Wehen habe. |  |  | 0,510 | -0,093 | 0,126 | When I arrived at the maternity clinic, they believed that my birth was in progress/ I was having contractions. |  |
| Klinik_3 | In der Geburtsklinik standen genügend Betten, Zimmer, Kreißsäle oder Operationssäle für die Anzahl der Gebärenden zur Verfügung. |  |  | 0,700 | -0,106 | -0,269 | The maternity clinic had enough beds, rooms, delivery rooms or operating theaters for the number of women giving birth. |  |
| Klinik_4 | In der Geburtsklinik waren genügend Hebammen, ärztliche Fachkräfte und Pflegepersonal im Dienst, um alle Gebärenden versorgen zu können. |  |  | 0,737 | -0,116 | -0,369 | There were enough midwives, doctors and nursing staff on duty at the maternity clinic to care for all the women giving birth. |  |
| Klinik_5 | In der Geburtsklinik war es sauber und es standen ausreichend Desinfektionsspender, Seife und Waschbecken zur Benutzung bereit. |  |  | 0,522 | 0,160 | -0,081 | The maternity clinic was clean and there were plenty of disinfectant dispensers, soap and washbasins available for use. |  |
| Klinik_6 | In der Geburtsklinik gab es ausreichend Sichtschutz, so dass ich vor den Blicken unbeteiligter Personen geschützt war (z.B. durch Vorhänge, Raumteiler, Position des Betts im Raum, Laken). |  |  | 0,484 | 0,263 | -0,041 | There was sufficient privacy in the maternity clinic so that I was protected from the view of uninvolved persons (e.g. by curtains, room dividers, position of the bed in the room, sheets). |  |
| Klinik_7 | In der Geburtsklinik gab es Hilfmittel, die ich während der Wehen nutzen konnte (z.B. Gymnastikball, Sprossenwand, Geburtstuch, Geburtswanne, Geburtshocker). |  |  | 0,504 | 0,009 | 0,034 | In the maternity clinic there were aids that I could use during labor (e.g. exercise ball, wall bars, birth sling, birth tub, birth stool). |  |
| Klinik_8 | In der Geburtsklinik verbrachte ich während der Geburt längere Zeit unfreiwillig mit unbeteiligten Personen oder in einem öffentlichen Bereich (z.B. Flur, Wartebereich, andere Patient*innen, Besucher*innen von Mitpatient*innen). |  | x | 0,527 | 0,080 | 0,039 | In the maternity clinic, I spent long periods of time involuntarily with uninvolved persons or in a public area (e.g. corridor, waiting area, other patients, visitors of fellow patients) during the birth. |  |
| *Definition der Richtung: Je höher der Wert, desto positiver die Erfahrungen mit der geburtshilflichen Einrichtung* | | | | | | | *Definition of direction: The higher the value, the more positive the experience with the obstetric facility* | |
|  |  |  |  |  |  |  |  |  |
| **Construct: CARE AVAILABILITY** | | | | | | |  |  |
| **Variable** | | | **Inverted** | **Factor** | | | *English translation (not validated)* | |
|  |  |  |  | **1** | **2** |  |  |  |
| Betr_1 | Eine Hebamme blieb so lange bei mir, wie ich brauchte. | 1 trifft gar nicht zu 2 trifft eher nicht zu 3 trifft teilweise zu 4 trifft eher zu 5 trifft voll zu  0 kann/ möchte ich nicht beantworten |  | 0,835 | -0,192 |  | A midwife stayed with me for as long as I needed. | 1 Does not apply at all  2 rather not applicable  3 partly true  4 Rather true  5 fully applies  0 I cannot/would not like to answer |
| Betr_2 | Eine Hebamme kam regelmäßig zu mir, um sich nach meinem Befinden zu erkundigen. |  |  | 0,837 | -0,244 |  | A midwife came to see me regularly to check on my condition. |  |
| Betr_3 | Eine Hebamme kam zeitnah zu mir, wenn ich nach ihr fragte/klingelte und kümmerte sich um meine Anliegen. |  |  | 0,845 | -0,187 |  | A midwife came to me promptly when I asked for her/ rang the bell and took care of my concerns. |  |
| Betr_4 | Eine ärztliche Fachkraft (für Gynäkologie und Geburtshilfe, Allgemeinmediziner*in, Anästhesist*in) war immer, wenn ich Bedarf nach medizinischer Betreuung äußerte, für mich da. |  |  | 0,723 | 0,050 |  | A doctor (for gynecology and obstetrics, general practitioner, anesthesiologist) was always there for me when I expressed a need for medical care. |  |
| Betr_5 | Eine Hebamme war in der letzten Phase vor der Geburt meines Kindes (Austreibungsphase, meist mit Presswehen) oder in den 20 Minuten, bevor die Entscheidung zum Kaiserschnitt getroffen wurde, durchgängig in meiner Nähe. |  |  | 0,645 | 0,290 |  | A midwife was always nearby in the last phase before the birth of my child (expulsion phase, usually with pushing contractions) or in the 20 minutes before the decision was made to have a caesarean section.  decision to have a caesarean section was made. |  |
| *Definition der Richtung: Je höher der Wert, desto positiver die Erfahrung mit der geburtshilflichen Betreuung* | | | | | | | *Definition of direction: the higher the value, the more positive the experience with obstetric care* | |
|  |  |  |  |  |  |  |  |  |
| **Construct: INFORMATION** | | | | | | | *English translation (not validated)* |  |
| **Variable** | | | **Inverted** | **Factor** | | |  |  |
|  |  |  |  | **1** |  |  |  |  |
| Info_1 | Ich erhielt widersprüchliche, unklare oder falsche Informationen zum Geburtsverlauf oder dem Gesundheitszustand von mir/ meinem Kind. | 1 trifft gar nicht zu 2 trifft eher nicht zu 3 trifft teilweise zu 4 trifft eher zu 5 trifft voll zu  0 kann/ möchte ich nicht beantworten | x | .633 |  |  | I received contradictory, unclear or incorrect information about the birth process or the state of health of me/my child. | 1 Does not apply at all  2 rather not applicable  3 partly true  4 Rather true  5 fully applies  0 I cannot/would not like to answer |
| Info_2 | Nach Untersuchungen wurde ich über die Befunde (z.B. Öffnung des Muttermunds) und die Bedeutung für den weiteren Geburtsverlauf ausreichend informiert. |  |  | .765 |  |  | After examinations, I was sufficiently informed about the findings (e.g. opening of the cervix) and the significance for the further course of the birth. |  |
| Info_3 | Die Hebamme nahm sich ausreichend Zeit für Erklärungen oder um meine Fragen zu beantworten. |  |  | .783 |  |  | The midwife took enough time to explain things and answer my questions. |  |
| Info_4 | Die Hebamme kommunizierte mit mir in einer Sprache, die ich verstehen konnte (Erklärung von Fachbegriffen, Verwendung einfacher Sprache). |  |  | .636 |  |  | The midwife communicated with me in a language I could understand (explaining technical terms, using simple language). |  |
| Info_5 | Die ärztlichen Fachkräfte nahmen sich ausreichend Zeit für Erklärungen oder um meine Fragen zu beantworten. |  |  | .773 |  |  | The doctors took enough time to explain things and answer my questions. |  |
| Info_6 | Die ärztlichen Fachkräfte kommunizierten mit mir in einer Sprache, die ich verstehen konnte (Erklärung von Fachbegriffen/ Befunden, Verwendung einfacher Sprache). |  |  | .651 |  |  | The doctors communicated with me in a language I could understand (explanation of technical terms/findings, use of simple language). |  |
| *Definition der Richtung: Je höher der Wert, desto positiver die Erfahrungen mit der Vermittlung von Information/ Kommunikation.* | | | | | | | *Definition of direction: The higher the value, the more positive the experience with the provision of information/communication.* | |
|  |  |  |  |  |  |  |  |  |
| **Construct: CONSENT** | | | | | | |  |  |
| **Variable** | | | **Inverted** | **Factor** | | | *English translation (not validated)* | |
|  |  |  |  | **1** |  |  |  |  |
| Consent_1 | Wurden Sie während der Geburt zu einer geburtshilflichen Intervention gedrängt? | 1 trifft gar nicht zu 2 trifft eher nicht zu 3 trifft teilweise zu 4 trifft eher zu 5 trifft voll zu  0 kann/ möchte ich nicht beantworten | x | 0,703 |  |  | Were you pressured into an obstetric intervention during the birth? | 1 Does not apply at all  2 rather not applicable  3 partly true  4 Rather true  5 fully applies  0 I cannot/would not like to answer |
| Consent_2 | Wurden Sie während der Geburt zu einer Gebärposition gedrängt? |  | x | 0,690 |  |  | Were you forced into a birthing position during the birth? |  |
| Consent_3 | Hatten Sie während der Geburt das Gefühl, Interventionen nicht ablehnen zu können, da sie als alternativlos dargestellt wurden und/oder Sie negative Konsequenzen fürchteten? |  | x | 0,842 |  |  | Did you have the feeling during the birth that you could not refuse interventions because they were presented as having no alternative and/or you feared negative consequences? |  |
| *Definition der Richtung: Je höher der Wert, desto positiver die Erfahrung mit Aufklärung und Einwilligung.* | | | | | | | *Definition of direction: The higher the value, the more positive the experience with information and consent.* | |
|  |  |  |  |  |  |  |  |  |
| **Construct: PRIVACY** | | | | | | |  |  |
| **Variable** | | | **Inverted** | **Factor** | | | *English translation (not validated)* | |
|  |  |  |  | **1** |  |  |  |  |
| Privat_1 | Die geburtshilflichen Fachkräfte schlossen die Tür zu meinem Raum immer zügig, so dass diese nie länger als notwendig offenstand. | 1 trifft gar nicht zu 2 trifft eher nicht zu 3 trifft teilweise zu 4 trifft eher zu 5 trifft voll zu  0 kann/ möchte ich nicht beantworten |  | 0,704 |  |  | The maternity care staff always closed the door to my room quickly so that it was never open for longer than necessary. | 1 Does not apply at all  2 rather not applicable  3 partly true  4 Rather true  5 fully applies  0 I cannot/would not like to answer |
| Privat_2 | Die geburtshilflichen Fachkräfte gingen respektvoll damit um, dass ich (teilweise) unbekleidet war. |  |  | 0,709 |  |  | The maternity care staff were respectful of the fact that I was (partially) unclothed. |  |
| Privat_3 | Die geburtshilflichen Fachkräfte sorgten dafür, dass keine unbeteiligten Personen anwesend waren, wenn ich körperlich untersucht, behandelt oder beraten wurde. |  |  | 0,820 |  |  | The maternity care staff made sure that no uninvolved persons were present when I was being physically examined, treated or advised. |  |
| Privat_4 | Die geburtshilflichen Fachkräfte sorgten dafür, dass Gespräche zwischen mir und ihnen nicht durch Unbeteiligte mitgehört werden konnten. |  |  | 0,865 |  |  | The maternity care staff made sure that conversations between me and them could not be overheard by bystanders. |  |
| Privat_5 | Die geburtshilflichen Fachkräfte sorgten dafür, dass keine Unbeteiligten mithören konnten, während sie sich über mich, meinen Geburtsfortschritt oder meine gesundheitsbezogenen Informationen austauschten. |  |  | 0,917 |  |  | The maternity care staff made sure that no bystanders could overhear them talking about me, my labor progress or my health-related information. |  |
| *Definition der Richtung: Je höher der Wert, desto positiver die Erfahrung mit dem Schutz der Privatsphäre.* | | | | | | | *Definition of direction: The higher the value, the more positive the experience of privacy protection.* | |
|  |  |  |  |  |  |  |  |  |
| **Construct: SUPPORT** | | | | | | |  |  |
| **Variable** | | | **Inverted** | **Factor** | | | *English translation (not validated)* | |
|  |  |  |  | **1** |  |  |  |  |
| Support_1 | Die Hebamme respektierten meine Wünsche und Vorstellungen für die Geburt und setzte diese (wenn möglich) um. | 1 trifft gar nicht zu 2 trifft eher nicht zu 3 trifft teilweise zu 4 trifft eher zu 5 trifft voll zu  0 kann/ möchte ich nicht beantworten |  | 0,850 |  |  | The midwife respected my wishes and ideas for the birth and realized them (if possible). | 1 Does not apply at all  2 rather not applicable  3 partly true  4 Rather true  5 fully applies  0 I cannot/would not like to answer |
| Support_2 | Die Hebamme hat mich aktiv in Entscheidungsprozesse eingebunden. |  |  | 0,855 |  |  | The midwife actively involved me in decision-making processes. |  |
| Support_3 | Die Hebamme hörte mir zu, ging einfühlsam auf meine Ängste, Sorgen und Schmerzen ein und war wohlwollend mit mir. |  |  | 0,811 |  |  | The midwife listened to me, responded sensitively to my fears, worries and pain and was kind to me. |  |
| Support_4 | Die Hebamme zeigte mir Möglichkeiten auf, Wehen- und Geburtsschmerzen zu bewältigen (Beispiele: Atemtechniken, Bewegungen, Positionen, Akupressur, Wanne). |  |  | 0,767 |  |  | The midwife showed me ways to cope with labor and birth pain (examples: breathing techniques, movements, positions, acupressure, tub). |  |
| Support_5 | Die Hebamme ließ mir während der Geburt genügend Zeit/ räumte dem Geburtsprozess genügend Zeit ein. |  |  | 0,825 |  |  | The midwife gave me enough time during the birth/allowed enough time for the birth process. |  |
| Support_6 | Die ärztliche Fachkraft respektierte meine Wünsche und Vorstellungen für die Geburt und setzte diese (wenn möglich) um. |  |  | 0,861 |  |  | The doctors respected my wishes and ideas for the birth and implemented them (if possible). |  |
| Support_7 | Die ärztliche Fachkraft hat mich aktiv in Entscheidungsprozesse eingebunden. |  |  | 0,838 |  |  | The doctors actively involved me in decision-making processes. |  |
| Support_8 | Die ärztliche Fachkraft hörte mir zu, ging einfühlsam auf meine Ängste, Sorgen und Schmerzen ein und war wohlwollend mit mir. |  |  | 0,824 |  |  | The doctors listened to me, responded sensitively to my fears, worries and pain and was sympathetic towards me. |  |
| Support_9 | Die ärztliche Fachkraft ließ mir während der Geburt genügend Zeit/ räumte dem Geburtsprozess genügend Zeit ein. |  |  | 0,852 |  |  | The doctors gave me enough time during the birth/allowed enough time for the birth process. |  |
| *Definition der Richtung: Je höher der Wert, desto positiver die Erfahrungen mit Unterstützung durch geburtshilfliche Fachkräfte* | | | | | | | *Definition of direction: the higher the value, the more positive the experience of support from medical professionals* | |
|  |  |  |  |  |  |  |  |  |
| **Construct: RIGHTS** | | | | | | |  |  |
| **Variable** | | | **Inverted** | **Factor** | | | *English translation (not validated)* | |
|  |  |  |  | **1** | **2** |  |  |  |
| Rechte_1 | Auf meine kulturellen oder religiösen Wünsche und Bedürfnisse wurde Rücksicht genommen, sofern aus medizinischen Gründen nichts dagegensprach. | 1 trifft gar nicht zu 2 trifft eher nicht zu 3 trifft teilweise zu 4 trifft eher zu 5 trifft voll zu  0 kann/ möchte ich nicht beantworten |  | 0,750 | 0,449 |  | My cultural or religious wishes and needs were taken into consideration, unless there were medical reasons not to do so. | 1 Does not apply at all  2 rather not applicable  3 partly true  4 Rather true  5 fully applies  0 I cannot/would not like to answer |
| Rechte_2 | Ich durfte während der Geburt Essen oder Trinken zu mir nehmen. |  |  | 0,590 | 0,039 |  | I was allowed to eat or drink during the birth. |  |
| Rechte_3 | Ich durfte mich während der Eröffnungsphase der Geburt frei bewegen.  Beispiele: laufen, knien, verschiedene Positionen einnehmen |  |  | 0,723 | -0,271 |  | I was allowed to move freely during the opening phase of the birth. Examples: walking, kneeling, assuming different positions |  |
| Rechte_4 | Ich durfte mich während der Austreibungsphase der Geburt frei bewegen/ eine selbstgewählte Position einnehmen.  Austreibungsphase: letzte Phase der Geburt, meist mit Presswehen. |  |  | 0,571 | -0,373 |  | During the expulsion phase of the birth, I was allowed to move freely/ take up a position of my own choosing.  Expulsion phase: last phase of the birth, usually with pushing contractions. |  |
| *Definition der Richtung: Je höher der Wert, desto positiver die Erfahrung mit der Einhaltung von Rechten.* | | | | | | | *Definition of direction: The higher the value, the more positive the experience with the observance of rights.* | |
|  |  |  |  |  |  |  |  |  |
| **Construct: RESPECT** | | | | | | | *English translation (not validated)* | |
| **Variable** | | | **Inverted** | **Factor** | | |  |  |
|  |  |  |  | **1** | **2** | **3** |  |  |
| Respekt _1 | Die Hebammen haben auf freundliche, respektvolle und wohlwollende Weise mit mir kommuniziert (verbal und nonverbal). | 1 trifft gar nicht zu 2 trifft eher nicht zu 3 trifft teilweise zu 4 trifft eher zu 5 trifft voll zu  0 kann/ möchte ich nicht beantworten |  | 0,811 | -0,491 | -0,013 | The midwives communicated with me in a friendly, respectful and benevolent way (verbally and non-verbally). | 1 Does not apply at all  2 rather not applicable  3 partly true  4 Rather true  5 fully applies  0 I cannot/would not like to answer |
| Respekt _2 | Die ärztliche Fachkraft hat auf freundliche, respektvolle und wohlwollende Weise mit mir kommuniziert (verbal und nonverbal). |  |  | 0,706 | 0,047 | 0,234 | The obstetrician communicated with me in a friendly, respectful and benevolent manner (verbally and non-verbally). |  |
| Respekt _3 | Die Hebammen haben mich rücksichtsvoll und behutsam behandelt. |  |  | 0,779 | -0,502 | 0,013 | The midwives treated me with consideration and care. |  |
| Respekt _4 | Die geburtshilflichen Fachkräfte redeten, als sei ich nicht anwesend.  Beispiele: redeten über mich, statt mit mir, redeten über unangebrachte Themen. |  |  | 0,628 | 0,090 | 0,043 | The maternity care professionals talked as if I wasn't there.  Examples: talked about me instead of with me, talked about inappropriate topics. |  |
| Respekt _5 | Mindestens eine geburtshilfliche Fachkraft hat mich beleidigt, abgewertet, angeschrien, bloßgestellt oder unangemessen angesprochen. |  |  | 0,809 | 0,174 | -0,264 | At least one maternity care professional has insulted, belittled, shouted at, embarrassed or spoken to me inappropriately. |  |
| Respekt _6 | Mindestens eine geburtshilfliche Fachkraft hat mir Angst gemacht, mich eingeschüchtert oder mir gedroht. |  | x | 0,758 | 0,187 | -0,188 | At least one maternity care professional scared me, intimidated me or threatened me. |  |
| Respekt _7 | Mindestens eine geburtshilfliche Fachkraft hat mir Vorwürfe gemacht oder mir gezeigt, dass ich in ihren Augen versage. |  | x | 0,779 | 0,088 | -0,344 | At least one maternity care professional reproached me or showed me that I was failing in their eyes. |  |
| Respekt _8 | Mindestens eine geburtshilfliche Fachkraft hat mich gedemütigt oder entmenschlicht. |  | x | 0,767 | 0,148 | -0,154 | At least one maternity care professional humiliated or dehumanized me. |  |
| Respekt _9 | Mindestens eine geburtshilfliche Fachkraft hat mich geschlagen, getreten, gekniffen, geschubst oder grob angefasst. |  | x | 0,557 | 0,271 | 0,088 | At least one maternity care professional has hit, kicked, pinched, pushed or roughly touched me. |  |
| Respekt _10 | Mindestens eine geburtshilfliche Fachkraft hat mich aufs Bett gedrückt, mich gegen meinen Willen körperlich eingeschränkt, festgebunden oder fixiert. |  | x | 0,503 | 0,136 | 0,155 | At least one maternity care professional pushed me onto the bed, physically restricted me against my will, tied me down or restrained me. |  |
| *Definition der Richtung: Je höher der Wert, desto positiver die Erfahrungen mit Respekt.* | | | | | | | *Definition of direction: The higher the value, the more positive the experiences with respect.* | |
|  |  |  |  |  |  |  |  |  |
| **Construct: BIRTH INTEGRITY** | | | | | | | *English translation (not validated)* | |
| **Variable** | | | **Inverted** | **Factor** | | |  |  |
|  |  |  |  | **1** | **2** |  |  |  |
| BInt_1 | Während der Geburt fühlte ich mich geachtet, respektiert und/oder wertgeschätzt. | 1 trifft gar nicht zu 2 trifft eher nicht zu 3 trifft teilweise zu 4 trifft eher zu 5 trifft voll zu  0 kann/ möchte ich nicht beantworten |  | 0,854 | 0,151 |  | During birth, I felt valued, respected and/or appreciated. | 1 does not apply at all 2 does rather not apply 3 does partly apply 4 does rather apply 5 does fully apply   0 cannot/ would like not to answer |
| BInt_2 | Während der Geburt ließ ich Dinge über mich ergehen oder gab ich auf, obwohl ich innerlichen Widerstand spürte. |  | x | 0,792 | 0,090 |  | During birth, I put up with things or gave up, even though I felt inner resistance. |  |
| BInt_3 | Während der Geburt fühlte ich mich sicher, beschützt und/oder gut aufgehoben. |  |  | 0,892 | 0,086 |  | During birth, I felt safe, protected and/or in good hands. |  |
| BInt_4 | Während der Geburt fühlte ich mich zeitweise abgetrennt, abwesend, dissoziiert und/oder betrachtete mich von "Außen". Achtung: hiermit ist kein wünschenswerter, hormonbedingter 'Rausch' oder 'Benebeltsein' gemeint, sondern eine Stress-Reaktion, die auftreten kann, wenn man einen starken Kontrollverlust spürt. |  | x | 0,705 | -0,034 |  | During birth, I felt disconnected, at times absent, dissociated and/or viewed myself from 'on the outside'.  *Attention: this does not mean a desirable, hormone-induced "high" or " cloudiness but a stress reaction that can occur when you feel a strong loss of control. |  |
| BInt_5 | Während der Geburt fühlte ich mich herabgewürdigt, entwürdigt und/oder entmenschlicht. |  | x | 0,758 | 0,262 |  | I felt belittled, degraded and/or dehumanized. |  |
| BInt_6 | Während der Geburt erlebte ich eine Überschreitung meiner Grenzen durch andere.  Achtung: hiermit ist nicht gemeint, dass der Geburtsprozess als solcher die eigenen Grenzen (z.B. Schmerz) überschreiten kann, sondern die Wahrnehmung, dass andere Personen Grenzen überschritten haben. |  | x | 0,795 | 0,302 |  | During birth, I experienced my boundaries being violated by others. |  |
| BInt_7 | Während der Geburt war ich ganz bei mir, innerlich ruhig und konnte mich auf den Geburtsprozess einlassen. |  |  | 0,800 | -0,272 |  | During birth, I was completely with myself, internally calm, and able to engage in the birthing process. |  |
| BInt_8 | Während der Geburt habe ich starke Nervosität, Ängste, Verzweiflung und/oder Panik entwickelt. |  | x | 0,683 | -0,191 |  | During birth, I have developed severe nervousness, anxiety, despair and/or panic. |  |
| BInt_9 | Während der Geburt fühlte ich mich einsam, alleine, vernachlässigt, übersehen und/oder für unwichtig erachtet. |  | x | 0,796 | 0,020 |  | During birth, I felt lonely, alone, neglected, overlooked and/or considered unimportant. |  |
| BInt_10 | Während der Geburt fühlte ich mich gesehen, gehört und/ oder ernstgenommen. |  |  | 0,832 | 0,038 |  | During birth, I felt seen, heard and/or taken seriously. |  |
| BInt_11 | Während der Geburt spürte ich Scham. |  | x | 0,478 | 0,048 |  | During birth, I felt shame. |  |
| BInt_12 | Während der Geburt hatte ich ausreichend Kontrolle über die Situation, um Vertrauen in die Handlungen anderer zu haben.  Achtung: hiermit ist nicht die Kontrolle über die körperlichen Prozess der Geburt gemeint. |  |  | 0,868 | 0,013 |  | During birth, I had enough control over the situation to have confidence in the actions of others.   *Please note: this does not refer to control over the physical process of birth. |  |
| BInt_13 | Während der Geburt fühlte ich mich eingeschüchtert, sprachlos und/oder ausgeliefert. |  | x | 0,867 | 0,210 |  | During birth, I felt intimidated, speechless and/or at the mercy of others. |  |
| BInt_14 | Während der Geburt fühlte ich mich verletzt, angegriffen und/oder 'in die Ecke gedrängt'. |  | x | 0,817 | 0,344 |  | During birth, I felt harmed, attacked and/ or as if I was left with no choice. |  |
| BInt_15 | Während der Geburt fühlte ich mich belastet, betrübt und/oder traurig. |  | x | 0,797 | -0,002 |  | During birth, I felt burdened, distressed and/or sad. |  |
| BInt_16 | Während der Geburt ich mich wohl, glücklich und/oder durch die Geburtshormone 'berauscht'. |  |  | 0,710 | -0,345 |  | During birth, I felt comfortable, happy and/or exhilarated' by the birth hormones. |  |
| BInt_17 | Während der Geburt mache/machte ich mir Vorwürfe. |  | x | 0,739 | -0,074 |  | Looking back at birth, I reproach myself. |  |
| BInt_18 | Während der Geburt empfinde ich Dankbarkeit, Wohlbefinden und/oder Zufriedenheit. |  |  | 0,865 | -0,224 |  | Looking back at birth, I feel gratitude. well-being and/or satisfaction. |  |
| BInt_19 | Im Rückblick auf die Geburt empfinde ich Trauer, Enttäuschung und/oder (emotionalen) Schmerz. |  | x | 0,848 | -0,100 |  | Looking back at birth, I feel gratitude, a sense of well-being and/or satisfaction. |  |
| BInt_20 | Im Rückblick auf die Geburt empfinde ich Stress, Nervosität, Unruhe und/oder Reizbarkeit. |  | x | 0,812 | -0,100 |  | Looking back at birth, I feel stress, nervousness, anxiety and/or irritability. |  |
| BInt_21 | Im Rückblick auf die Geburt empfinde ich Stolz, Staunen und/oder Ergriffenheit. |  |  | 0,768 | -0,289 |  | Looking back at birth, I feel pride, amazement, and/or awe. |  |
| BInt_22 | Im Rückblick auf die Geburt empfinde ich Ärger, Wut oder Fassungslosigkeit. |  | x | 0,864 | 0,111 |  | Looking back at birth, I feel anger, rage and/or bewilderment. |  |
| *Definition der Richtung: Je höher der Wert, desto höher/ gewahrter die Geburtsintegrität* | | | | | | | *Definition of the direction: the higher the value, the higher/more preserved the birth integrity* | |
